# Assessing the benefits of five years of different approaches to treatment of urogenital schistosomiasis: A SCORE project in Northern Mozambique

**DOI:** 10.1371/journal.pntd.0006061

**Published:** 2017-12-08

**Authors:** Anna E. Phillips, Pedro H. Gazzinelli-Guimaraes, Herminio O. Aurelio, Josefo Ferro, Rassul Nala, Michelle Clements, Charles H. King, Alan Fenwick, Fiona M. Fleming, Neerav Dhanani

**Affiliations:** 1 Schistosomiasis Control Initiative, Department of Infectious Disease Epidemiology, Imperial College London, London, United Kingdom; 2 Faculdade of Health Sciences, Universidade Católica de Moçambique (UCM) Beira, Moçambique; 3 Laboratório de Parasitologia Intestinal e Vesical do Instituto Nacional de Saúde de Moçambique, Ministerio da Saúde, Maputo, Moçambique; 4 Center for Global Health and Diseases, School of Medicine, Case Western Reserve University, Euclid Avenue, Cleveland, Ohio, United States of America; 5 Schistosomiasis Consortium for Operational Research and Evaluation, University of Georgia, Athens, Georgia, United States of America; Swiss Tropical and Public Health Institute, SWITZERLAND

## Abstract

**Background:**

In Mozambique, schistosomiasis is highly endemic across the whole country. The Schistosomiasis Consortium for Operational Research and Evaluation (SCORE) coordinates a five-year study that has been implemented in various African countries, including Mozambique. The overall goal of SCORE was to better understand how to best apply preventive chemotherapy with praziquantel (PZQ) for schistosomiasis control by evaluating the impact of alternative treatment approaches.

**Methods:**

This was a cluster-randomised trial that compared the impact of different treatment strategies in study areas with prevalence among school children of ≥21% *S*. *haematobium* infection by urine dipstick. Each village was randomly allocated to one of six possible combinations of community-wide treatment (CWT), school-based treatment (SBT), and/or drug holidays over a period of four years, followed by final data collection in the fifth year. The most intense intervention arm involved four years of CWT, while the least intensive arm involved two years of SBT followed by two consecutive years of PZQ holiday. Each study arm included 25 villages randomly assigned to one of the six treatment arms. The primary outcome of interest was change in prevalence and intensity of *S*. *haematobium* among 100 children aged 9-to-12-years that were sampled each year in every village. In addition to children aged 9-to-12 years, 100 children aged 5–8 years in their first-year of school and 50 adults (aged 20–55 years) were tested in the first and final fifth year of the study. Prevalence and intensity of *S*. *haematobium* infection was evaluated by two filtrations, each of 10mL, from a single urine specimen.

**Principal findings:**

In total, data was collected from 81,167 individuals across 149 villages in ten districts of Cabo Delgado province, Northern Mozambique. Overall PZQ treatment resulted in a significant reduction in the prevalence of *S*. *haematobium* infection from Year 1 to Year 5, where the average prevalence went from 60.5% to 38.8%, across all age groups and treatment arms. The proportion of those heavily infected also reduced from 17.6% to 11.9% over five years. There was a significantly higher likelihood of males being infected than females at baseline, but no significant difference between the sexes in their response to treatment. The only significant response based on a study arm was seen in both the 9-to-12-year-old and first-year cross sections, where two consecutive treatment holidays resulted in a significantly higher final prevalence of *S*. *haematobium* than no treatment holidays. When the arms were grouped together, four rounds of treatment (regardless of whether it was CWT or SBT), however, did result in a significantly greater reduction in *S*. *haematobium* prevalence than two rounds of treatment (i.e. with two intermittent or consecutive holiday years) over a five-year period.

**Conclusions:**

Although PC was successful in reducing the burden of active infection, even among those heavily infected, annual CWT did not have a significantly greater impact on disease prevalence or intensity than less intense treatment arms. This may be due to extremely high starting prevalence and intensity in the study area, with frequent exposure to reinfection, or related to challenges in achieving high treatment coverage More frequent treatment had a greater impact on prevalence and intensity of infection when arms were grouped by number of treatments, however, cost efficiency was greater in arms only receiving two treatments. Finally, a significant reduction in prevalence of *S*. *haematobium* was seen in adults even in the SBT arms implying the rate of transmission in the community had been decreased, even where only school children have been treated, which has significant logistical and cost-saving implications for a national control programme in justifying CWT.

## Author summary

Urogenital schistosomiasis is highly endemic in Mozambique. This study was part of a multi-country trial, including Mozambique, designed to understand the impact of different schistosomiasis treatment strategies involving community-wide treatment (CWT), school-based (SBT), and treatment holidays over a five-year period. Results from Mozambique showed that although preventive chemotherapy was successful in reducing the prevalence of *Schistosoma haematobium* over five-years, the most intense treatment approach, annual CWT, did not have a significantly greater impact than less intense treatment strategies, such as bi-annual SBT. Infection rates were higher among males, but there was no difference in response to treatment by gender. Four rounds of treatment (regardless of whether it was given in the community or school) did result in a significantly greater reduction of *S*. *haematobium* prevalence than two rounds of treatment over a five-year period. There was, however, a resurgent increase in prevalence and intensity of *S*. *haematobium* infection shown after two consecutive treatment-holiday years, implying a bounce back in infection after a two year pause in treatment. Interestingly and unexpectedly, there was a significant reduction in prevalence of schistosomiasis in adults even in communities that had received SBT implying the force of transmission in the community had been decreased, even where only school children had been treated. These findings provide an evidence-base with significant logistical and cost-saving implications for programmatic decisions on how best to gain control of *Schistosoma haematobium*.

## Introduction

Schistosomiasis is a major yet neglected public health problem, second only to malaria in terms of parasite-induced human morbidity and mortality worldwide [1]. Estimates show that globally at least 218 million people required preventive treatment in 2015 [2–4], and at least 20 million suffer from severe and debilitating forms of the disease [5]. Schistosomiasis is a major public health problem in Mozambique, as shown by an epidemiological survey of schistosomiasis and soil-transmitted helminthiasis among school children carried out between 2005 and 2007 [6]. The mean estimated prevalence of urogenital schistosomiasis, *Schistosoma haematobium*, was 47% while that of intestinal schistosomiasis, *S*. *mansoni*, was much lower (around 1%) across all of Mozambique. In Cabo Delgado province, the area where this study took place, the prevalence of *S*. *haematobium* was 57.9%, ranging from 8.8% on the coast to 93% inland [6].

In 2001, the World Health Organisation (WHO) endorsed preventive chemotherapy (PC) as the global strategy to control morbidity due to schistosomiasis through regular treatment with praziquantel (PZQ) [7]. Treating all school-aged children (SAC) is more cost-effective than a test-and-treat approach [8, 9], which is achieved by allocating a geographic area (typically the district) to a recommended WHO treatment category for schistosomiasis based on infection prevalence [7]. As a result, most programmes control morbidity associated with schistosomiasis through school-based deworming, as it is highly cost-effective [10]. However, the limitation of this approach is poor coverage among out-of-school children, particularly older children and those living far from schools, and neglecting children younger than four years and adults [11]. Studies have highlighted the advantages of a community-wide treatment (CWT) approach in addition to a school-based treatment (SBT) in reducing prevalence and intensity of schistosomiasis infection, particularly in communities where not all children attend school [11–15]. Furthermore, questions remain about optimal frequency of PZQ treatment for infection and morbidity control. It has been observed in some studies that more frequent dosing can improve reductions in worm burden and increase parasitological cure, particularly where schistosomiasis is highly endemic [16]. Whereas others have shown that a single dose of PZQ results in sustained low transmission of *S*. *haematobium* for two years [17].

The Schistosomiasis Consortium for Operational Research and Evaluation (SCORE) was established in 2008 to answer strategic questions about schistosomiasis control [18]. It includes multi-country field studies that aim to understand the benefits and costs of alternative approaches to PC involving CWT, SBT and “drug holidays” (i.e. years without PC). The gaining and sustaining SCORE study protocol and baseline characteristics have been described elsewhere [18–21]. The primary research question presented here is which strategy for PC provided the greatest reduction in prevalence and intensity of *S*. *haematobium* infection among 9-to-12-year olds after four years of intervention in Cabo Delgado, Northern Mozambique. In addition, the impact of treatment on first-year students and adults sampled in each village was also assessed. These findings will provide an evidence base for the Mozambique national control programme for schistosomiasis to address strategic questions about schistosomiasis treatment and potentially shift from morbidity control to interruption of transmission, and subsequently elimination.

## Methods

### Study arms

**Fig 1:**
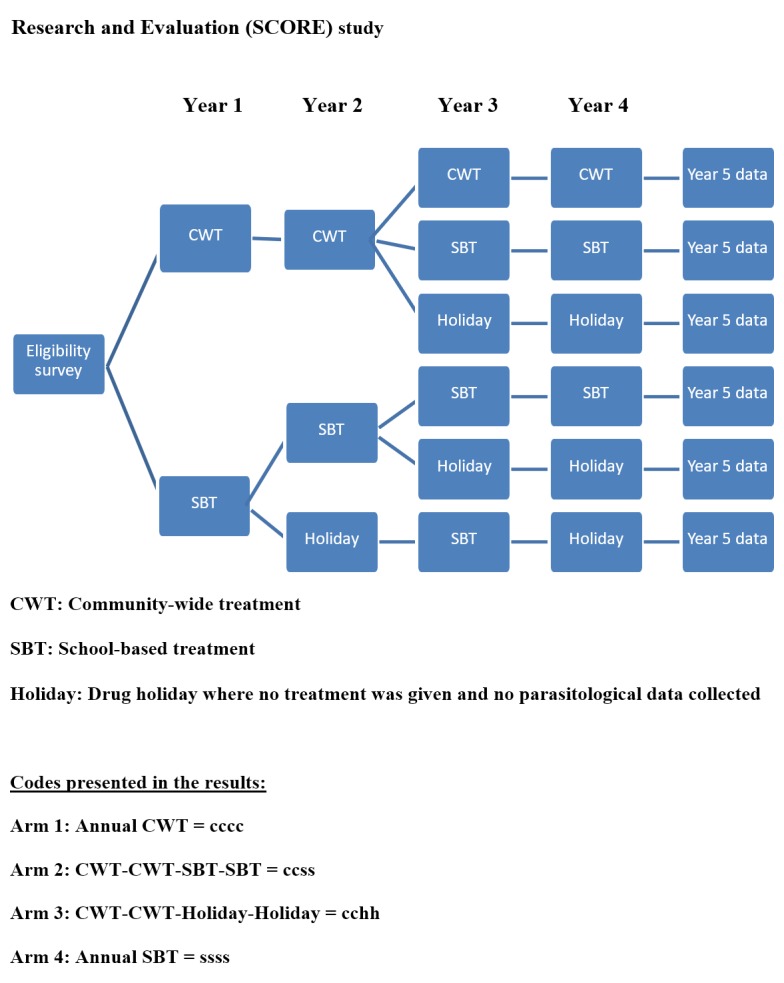
Study arms for gaining control of Schistosomiasis Consortium for Operational Research and Evaluation (SCORE) study [18]. There are six different potential study arms, where by each arm received a four-year treatment strategy with varying combinations of community-wide treatment (CWT), school-based treatment (SBT) and “drug holidays” (i.e. where no treatment is given): Arm 1: Annual CWT, Arm 2: CWT-CWT- SBT-SBT, Arm 3: CWT-CWT- Holiday-Holiday, Arm 4: Annual SBT, Arm 5: SBT-SBT-Holiday-Holiday, Arm 6: SBT-Holiday-SBT-Holiday.

This study was a parallel cluster-randomised, intervention trial with six study arms (Fig 1). Communities received various combinations of CWT, SBT or drug holiday over a four-year period, with the final round of data collection carried out 12 months after Year 4 treatment. All communities received CWT directly after Year 5 data collection, as they were no longer participating in the trial. The most intensive intervention arm involves four years of CWT (Arm 1), whilst the least intensive treatment strategy was two years of SBT PC followed by two consecutive years of drug holidays (Arm 5).

### Study area and population

The area for this study was 10 out of 17 districts of Mozambique’s northern most province, Cabo Delgado. The area was chosen as previous mapping had shown a high prevalence of *S*. *haematobium* and a low prevalence of *S*. *mansoni*, as one of the country selection criteria was no mixed infections to ensure that species type did not confound response to a particular treatment strategy [18].

Communities to be included in the study were determined by convenience sampling based on three criteria: (i) selecting only villages that had a primary school (so that it could be randomised to SBT), (ii) village and school had no history of PC using PZQ against schistosomiasis, (iii) and the village has a school attended by a minimum of 100 children aged 9-to-12-years. Given that children who test positive must be treated, the eligibility survey was carried out among fifty 13-to-14-year olds in each village, therefore treating those infected did not affect subsequent study results, especially where prevalence was high [18]. The starting prevalence of *S*. *haematobium* was evaluated by reagent strip testing for microhaematuria on a single mid-day urine (communities were eligible only if ≥21% by urine dipstick was detected) [22]. A total of 150 communities found eligible were randomly assigned using a computer-based randomisation procedure, without stratification, to one of the six study arms. Since the rainy season lasts from November to April, data were collected between July and October each year to omit seasonal variation. A door-to-door census was conducted prior to treatment in Year 1 in all selected communities to provide a baseline for estimating coverage.

### Sample size

The aim was to enrol 25 villages per study arm, and to monitor each village’s prevalence and intensity of *S*. *haematobium* infection among 100 school children aged 9-to-12-years. In each school, 50 boys and 50 girls were selected using systematic random sampling from those in the second, third and fourth classes. A total of 150 villages were enrolled, with 15,000 children tested each year. Parasitological data were collected prior to treatment from Year 1 through to Year 5. To evaluate the effect of treatment holidays, currently recommended by WHO PC guidelines whereby moderate-risk communities are treated once every two years [7], no testing of children was conducted during holiday years as children who were found to be infected would have needed to be treated. Sample size calculations assumed treatment interventions would reduce schistosomiasis prevalence in high endemicity areas from 50% in Year 1 to 15% by the end of the study in the most intense treatment (Arm 1). Power computations used generalized estimating equations to fit a logistic regression model that included treatment arm and time effects and treatment-by-time interaction, and assumed an over-dispersion parameter of φ = 5.0. The minimum effect size was computed so that a difference over time could be detected with 90% power for a 2-sided α = 0.05 level test. Negligible correlation was assumed between variables in Year 1 and at study conclusion. Based on these assumptions, the calculations estimated that studying 25 villages per arm and 100 children per village were sufficient to detect an overall difference between arms of 11.4% prevalence at the conclusion of the study. In addition to children aged 9-to-12-years, systematic random sampling was used in each village to select 100 first-year students (aged 5-to-8-years old) and a convenience sample of 50 adults (aged 20-to-55-years) in the first and fifth years only.

### Urine examination

In each school, selected children were given a plastic pot labelled with an adhesive barcode with unique identification numbers and asked to provide a single urine specimen. The sex, age, and school class for each child were recorded. All samples were collected between 10am and 2pm and examined immediately in the school. Two filtrations of 10mL each were carried out on a single urine sample using a nylon filter with a pore size of 20µm (Sefar AG; Heiden, Switzerland) [23, 24]. Both filters were examined for *S*. *haematobium* eggs under a light microscope and number of *S*. *haematobium* eggs were counted. All parasitological examinations were performed by trained laboratory technicians. For quality control, around 10% of all microscope slides were re-examined by a senior technician. The intensity of infection was expressed as the number of eggs per 10mL of urine filtered. For specimens of less than 10 mL, the volume of urine filtered was measured and the number of eggs per 10mL calculated. If estimated counts were above 1,000 eggs per 10mL, they were truncated at 1,000. The arithmetic mean of two filtrations taken from a single urine specimen was calculated to be the egg count of the child [25]. A child was deemed egg positive if one or more eggs were found in any of the slides examined.

### Treatment

Praziquantel tablets were delivered at approximately 40mg/kg using a WHO dose pole [26]. All treatment was carried out in the village on the same day as sampling whereby everyone was treated regardless of their parasitological results, which were anonymous. During SBT, directly-observed treatment with PZQ was administered by trained teachers to all children attending school. Efforts were made to treat non-school attendees through community sensitisation and mobilisation efforts. CWT involved providing treatment to the entire eligible population using community drug distributors either door-to-door or at fixed points in the study community, which only excluded children under 4 years of age or under 94 cm in height. Everyone who had taken the medication in SBT was monitored for adverse events for four hours after treatment, since the analysis was carried out in the school. In the CWT, since the drug distributor lives in the village they were available in the community.

At baseline (Year 1) a census was carried out in all selected communities of the total population and proportion of all SAC (aged 5-14-years) recorded in the population to provide a baseline for estimating coverage.

### Ethical statement

Informed consent was obtained from all individuals ≥18 years of age and from parents or legal guardians of children less than 18 years of age. The purpose of the study was explained to all schoolchildren and verbal assent was obtained from the children. Permission was also obtained from school headmasters. Ethical clearance was obtained from the National Bio-ethical Committee for Health of Mozambique (NBCHM), and the survey was conducted according to NBCHM guidelines (reference no. IRB00002657). The trial is registered with the International Standard Randomised Controlled Trial registry under ISRTC number 14117624 for Mozambique. The study protocol was also approved by Imperial College London (ICREC_10_2_2).

### Data handling and analysis

Demographic data were collected on smartphones and uploaded to a dedicated database maintained on a central server (EpiCollect at Imperial College London). Laboratory data were collected on paper forms and entered onto the smartphone retrospectively using the barcode with unique identification number to link with the individual’s demographic information. The electronic data capture system involved entering data in the field, synching the data to a central server and downloading the data retrospectively for cleaning and analysis. Data cleaning and management was carried out by biostatisticians at the Schistosomiasis Control Initiative (SCI). Data analysis was performed using R version 3.2 (R Core Team (2013): R: A language and environment for statistical computing. R Foundation for Statistical Computing, Vienna, Austria).

Since 9-to-12-year olds are the only age group to be consistently sampled from baseline to Year 5, the primary research question presented here is which strategy for PC provided the greatest reduction in prevalence and intensity of *S*. *haematobium* infection among 9-to-12-year olds after four years of intervention. In addition, the impact of treatment on first-year students and adults have also been reviewed and analysed.

Individuals without data on age, sex, and presence or absence of eggs on at least one slide were not included in the study. For each year of data, three infection categories for *S*. *haematobium* infection were created: no infection, light infection (<50 eggs/10mL of urine), and heavy infection (≥50 eggs/10mL of urine) [7]. Indicator variables were created for infection status (0 = not infected, 1 = infected) and heavy infection status (0 = egg count <50, 1 = egg count>50). These were averaged across each village to produce a village level prevalence and village level prevalence of heavy infected, respectively. 95% confidence intervals were calculated for village and study arm level prevalence values. *Survey* package [27] was used to consider survey design effects. Prevalence of infection and heavy infection was calculated as arithmetic means of the infection categories, aggregated by the relevant factors (e.g. age, sex). The arithmetic mean of infection intensity was calculated using both egg positive and negative, aggregated by grouping factors (village, study arm and gender). Although, mean intensity was underestimated as egg counts were capped at 1000 eggs/10mL, this was done consistently over the years therefore the trend in intensity change is believed to be valid. The egg reduction rate was calculated as the reduction in the intensity of infection assessed indirectly, using egg count via the following formula: % egg = 10mL reduction (1−arithmetric mean of eggs/10mL urine after treatment in Year 5) x 100 arithmetic mean of eggs = 10mL urine before treatment at Baseline.

To assess overall population-level programmatic impact on the prevalence of *S*. *haematobium* between study arms, as per the standard analysis plan, agreed prior to analysis, Generalised Estimated Equations (GEE) were used to estimate the differences between arms in year 5 only; unadjusted estimates using only village and study arm as covariates and adjusted estimates including sex, age and weighting for number of children who provided data were modelled using SAS software 9.1.2. (SAS Institute Inc., Cary, NC, USA). The models were run on the three cross-sections of data: 9-to-12-year olds, first-year students (5-to-8-year olds), and adults (20-to-55-year olds). Prevalence was modelled using a binomial GEE with logit link function with Village IDs treated as the repeated measure and ‘Lsmestimate’ used to test pre-specified differences between arms. In some villages, the enrollment was less than the target study population size. To account for this, a village-weight term was added to the GEE model to weight results according to numbers of children tested per village. Mean number of eggs per 10mL urine (mean intensity of infection) was modelled with negative-binomial GEE and log link function. To avoid issues of multiple testing we focused on specific arm comparisons. These comparisons were: Arm 1 (cccc) vs Arm 2 (ccss), Arm 1 (cccc) vs Arm 3 (cchh), Arm 1 (cccc) vs Arm 4 (ssss), Arm 4 (ssss) vs Arm 5 (sshh), and Arm 4 (ssss) vs Arm 6 (shsh) (Fig 1).

To assess the significance of the observed reduction in prevalence of *S*. *haematobium* in adults in the SBT arms (4, 5, and 6) we performed a binomial Generalised Linear Mixed Model (GLMM) with logit link function with infection status (0/1) as the response, age (standardised), sex, study arm and study year as fixed effects and village as a random effect on a subset of the parasitological data including only adults in the school based study arms for Year 1 and Year 5.

Nominal treatment coverage stipulated in the study protocol was 75%. In practice this was not achieved in many places. Despite additional data collection and considerable analytical effort, we were unable to adequately validate coverage; for this reason further analysis incorporating treatment coverage would be inappropriate. As the issues appear to be consistent throughout the study villages, the randomised allocation of villages to study arms means between arm comparisons could still have validity.

Kulldorff’s space-time scan statistics were used to detect high-risk spatial clusters for schistosomiasis infection using the SatScan software version 9.1.1. (Information Management Services, Inc., Boston, MA, USA; www.satscan.org) [28, 29]. To detect the high-risk spatial clusters of cases, the statistics use a moving elliptical window scanning the study area, and the maximum size of which is no more than 50% of the total population. We reported statistically significant clusters with an indicated p value <0.05. The SaTScan program was used to obtain reported observed cases, expected cases, relative risk of infection, and locations of specific cluster.

## Results

Data were collected in the dry season between July and October each year from 2011 until 2015 in ten districts of Cabo Delgado province, Northern Mozambique. It was intended to sample 105,000 people from a total of 150 villages over the five years, but due to poor school enrolment and absence from many villages by children and adults working in the fields and mines, our final analysis included 81,167 individuals (77.3% participation). One village dropped out of the study prior to data collection due to political issues, therefore data is available from 149 villages.

**Fig 2:**
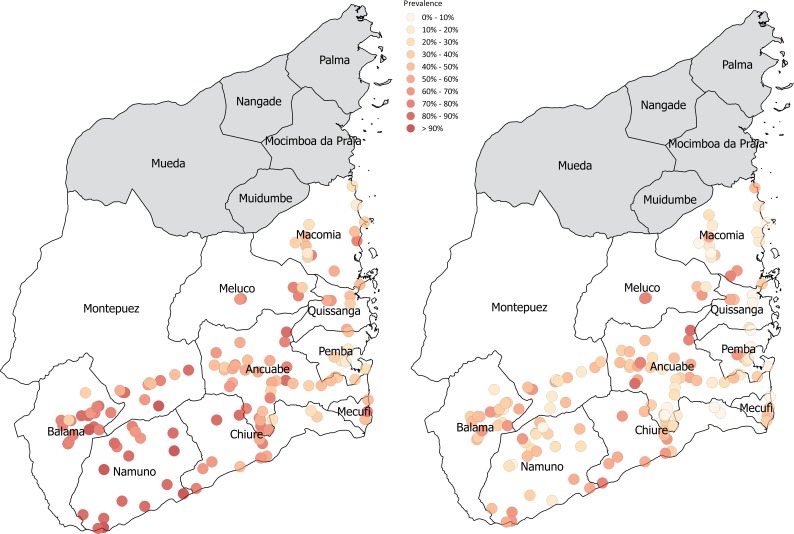
*S*. *haematobium* village-level prevalence across the ten survey districts in Cabo Delgado at baseline and Year 5. The *S*. *haematobium* prevalence of each village has been colour coded on the map according to level of prevalence. At baseline there are a greater number of higher prevalence (coded dark red), particularly in those areas furthest from the coast. At Year 5, the prevalence levels of each village have been reduced (coded yellow and orange) across all districts and areas of the map. We created the map ourselves using QGIS and publicly available shapefiles from http://www.diva-gis.org/gdata" to the figure caption in the manuscript.

Change in village-level prevalence from baseline to Year 5 is shown in Fig 2 and the SatScan in S1 Fig. Overall, forty-three percent of the population was female. Prevalence of infection decreased from baseline (60.5%) to Year 5 (38.8%), with prevalence of heavy infection also falling from 17.6% at baseline to 11.9% in Year 5 (Table 1). The change in prevalence of infection across each individual community over time by study arm has been summarised in the S2 Fig. Mean intensity of infection decreased from Year 1 to Year 2 but increases by Year 5. It appears therefore that although the number of individuals infected reduces over time, the burden of infection among infected individuals seem to be heavier. Study sample characteristics are summarised in Table 1 for gender, prevalence and intensity of infection across all age groups from baseline to Year 5.

**Table 1:** Sample characteristics by study year for all individuals (first-year students, 9-12-year olds, and adults inclusive) examined.

Year	No. of individuals sampled	Proportion female (%)	No. infected with *S*. *haematobium* (%)	No. with heavy intensity of infection (%)	Arithmetic mean *
1 (2011)	21,571	7,866 (36.5)	13,049 (60.5)	3,796 (17.6)	51.3
2 (2012)	10,424	4,291 (41.2)	5,086 (48.8)	1,157 (11.1)	33.8
3 (2013)	8,632	4,922 (57.0)	3,569 (41.4)	514 (5.95)	36.8
4 (2014)	7,174	3,224 (45.0)	2,985 (41.6)	776 (10.8)	37.3
5 (2015)	33,366	12,291 (36.8)	12,937 (38.8)	3,961 (11.9)	53.0

* This is the mean egg count among egg-positive subjects tested in each year of the study, which is an estimate of the intensity of infection among known active cases at each year of the study.

### 9-to-12-year old cross-section

Over five years, 42,731 independent observations were taken of children aged 9-to-12 years from 149 communities randomly allocated across six study arms. See S1 Table for a summary of *S*. *haematobium* infection rates for each study year and study arm (9-to-12-year olds only). The results in Table 2 and Fig 2 demonstrate the impact of treatment strategy on the prevalence and proportion of heavy infection of *S*. *haematobium* for the 9-to-12-year olds at baseline and Year 5. The initial prevalence of *S*. *haematobium* infection was 66.7% across all study arms for this age group, with 22.8% prevalence of heavy intensity of infection. Our findings show a reduction in Year 5 where *S*. *haematobium* infection decreased to 42.5% over all treatment arms and the proportion of those heavily infected reduced to 13.4%. The arithmetic mean intensity of infection at the village level, reduced over the course of the study from 69.1 eggs per 10ml urine to 58.1 eggs per 10ml, with changes in mean intensity between the study arms. The egg reduction rate from baseline to Year 5 was 13.5% (Table 2).

**Table 2:** Summary of *S*. *haematobium* infection from Baseline to Year 5 and study arm (9-to-12-year cross-section only).

Variable	Arm 1 (cccc)	Arm 2 (ccss)	Arm 3 (cshh)	Arm 4 (ssss)	Arm 5 (sshh)	Arm 6 (shsh)	Total/ Average
No. tested at baseline	1,270	1,081	1,208	1,421	1,287	962	7,229
No. infected at baseline	895	720	746	877	885	678	4,801
Prevalence at baseline (%)	70.5	66.6	61.8	61.7	68.8	70.5	66.7
Prevalence of heavy infection at baseline (%)	24.8	21.8	20.6	17.5	23.8	28.5	22.8
No. tested at Year 5	2,329	2,345	2,112	2,346	2,326	2,168	13,626
No. infected at Year 5	882	938	989	892	1210	871	5,782
Prevalence at Year 5 (%)	37.9	40.0	46.8	38.0	52.0	40.2	42.5
Prevalence of heavy infection at Year 5 (%)	10.4	11.9	15.9	13.1	17.5	11.6	13.4
Absolute difference between prevalence at Year 5 and baseline	-32.6	-26.6	-15.0	-23.7	-16.8	-30.3	-24.2
Relative difference between prevalence at Year 5 and baseline (% change)	-46.2	-39.9	-24.3	-38.4	-24.4	-43.0	-36.0
Village-level arithmetic mean infection intensity at baseline*	77.8	77.0	71.9	47.3	70.9	69.8	69.1
Village-level arithmetic mean infection intensity at Year 5*	40.5	53.3	71.9	55.2	77.0	50.3	58.1
Egg reduction rate (1-Year 5 intensity/baseline)	47.9%	30.8%	-0.1%	-16.9%	-8.6%	28.0%	13.5%
Individual-level arithmetic mean infection intensity at baseline**	81.6	79.5	80.4	52.9	63.8	80.8	73.2
Individual-level arithmetic mean infection intensity at Year 5 **	39.0	67.7	76.2	68.4	81.5	69.1	67.0

* Village-level intensity: This is the mean egg count for all tested 9-12-year-old subjects (including those with zero egg counts), which is a measure of community level contamination potential

** Individual-level intensity: This is the mean egg count among egg-positive subjects, which is an estimate of the intensity of infection among known active cases

cccc = annual CWT; ccss = CWT-CWT-SBT-SBT; cchh = CWT-CWT-Holiday-Holiday; ssss = Annual SBT; sshh = SBT-SBT-Holiday-Holiday; shsh = SBT-Holiday-SBT-Holiday

**Fig 3:**
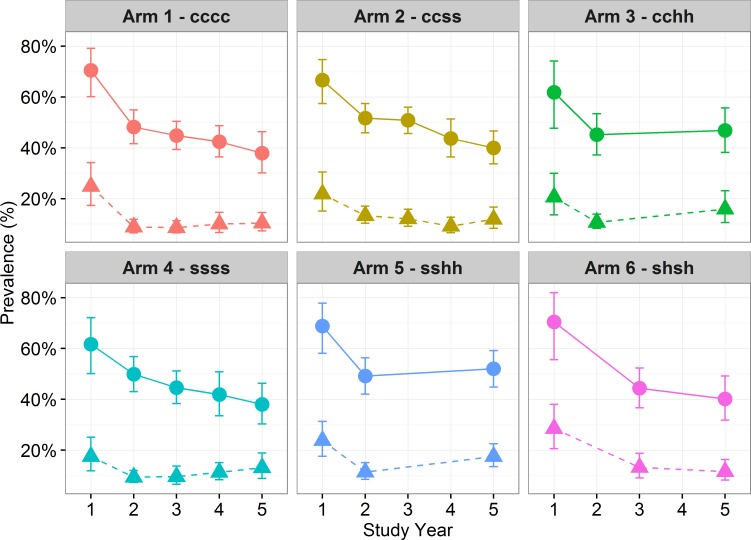
Prevalence of *S*. *haematobium* infection and heavy infection (dashed) by treatment over time (9–12 year-old-cross section only) and by study arm with 95% CI. Each graph shows the change in prevalence of *S*. *haematobium* infection at each time point from baseline to Year 5 by study arm, with heavy intensity of infection also marked on the graph. Prevalence and heavy infection levels decrease over time in all arms, with the exception of arm 3 and 5 following two consecutive holiday years where infection levels appear to increase where communities were not treated for a two year period.

Fig 3 shows the change in overall prevalence over time from the start of the project at baseline to the final Year 5 in each community across the 10 districts of the study. In both Arms 3 (cchh) and 5 (sshh) there are two consecutive years of a break in treatment (“Holiday”) in Years 3 and 4, following which there was an increase in prevalence and heavy intensity.

**Fig 4:**
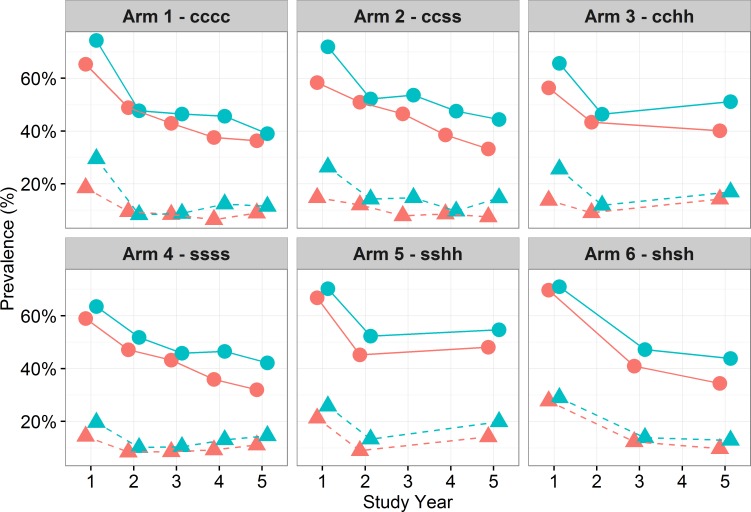
Prevalence of *S*. *haematobium* infection and heavy infection (dashed) by treatment by gender (males = blue and females = red) over time and study arm for 9–12 year-old-cross section. Each graph shows the change in prevalence of *S*. *haematobium* infection at each time point from baseline to Year 5 by study arm, with heavy intensity of infection also marked on the graph, separated by gender. There was no difference in response to treatment by gender, with both boys and girls’ prevalence and heavy infection levels decreasing over time in all arms, with the exception of arm 3 and 5 following two consecutive holiday years where infection levels increase.

Prevalence by gender demonstrated a higher prevalence of infection, as well as a greater proportion of those heavily infected, among males (p<0.001) (Table 3). Response to treatment was similar by gender, with both males and females showing reduced infection over time across all arms, except for Arms 3 and 5 (Fig 4). Thus, prevalence and intensity of infection appears to have increased after the treatment hiatus. The effects of treatment were analysed using Arm 1, the most frequent and wide treatment approach, as the reference case. Arm 1 (annual CWT), had a comparable impact on prevalence as the less intense Arm 6 (SBT every two years). Arm 1 had a greater reduction on prevalence than Arm 2, 3 and 4 across all years, although the difference was not statistically significant (Table 3). Arm 1 did have a significantly larger impact on *S*. *haematobium* infection than Arm 5 in Year 5 (p<0.024).

**Table 3:** GEE logistic model to assess differences in prevalence and intensity of *S*. *haematobium* infection between Year 1 and Year 5 and between study arms for each cross-section (33,459 Observations).

		Difference in Prevalence unadjusted		Difference in Prevalence adjusted		Difference in Intensity unadjusted		Difference in Intensity adjusted	
Age group	Arm comparison	Estimate (95% CI)	Pr > |z|	Estimate (95% CI)	Pr > |z|	Estimate (95% CI)	Pr > |z|	Estimate (95% CI)	Pr > |z|
9-to-12-year olds	arm 2 vs. arm 1	0.054 (-0.385,0.493)	0.809	0.08 (-0.365,0.526)	0.724	0.49 (-0.148,1.128)	0.132	0.446 (-0.273,1.166)	0.224
	arm 3 vs. arm 1	0.351 (-0.154,0.856)	0.173	0.372 (-0.161,0.905)	0.172	0.668 (0.073,1.263)	**0.028**	0.765 (0.099,1.432)	**0.025**
	arm 4 vs. arm 1	-0.022 (-0.512,0.469)	0.931	0.015 (-0.474,0.503)	0.953	0.507 (-0.189,1.202)	0.153	0.485 (-0.306,1.276)	0.229
	arm 5 vs. arm 4	0.547 (0.085,1.009)	**0.020**	0.547 (0.073,1.021)	**0.024**	0.273 (-0.409,0.954)	0.433	0.274 (-0.41,0.959)	0.432
	arm 6 vs. arm 4	0.127 (-0.401,0.655)	0.637	0.115 (-0.471,0.701)	0.701	-0.03 (-0.734,0.674)	0.934	-0.352 (1.127,0.423)	0.374
20-to-55-year olds	arm 2 vs. arm 1	0.096 (-0.325,0.516)	0.656	0.162 (-0.594,0.919)	0.674	-0.325 (1.034,0.384)	0.368	-0.24 (-0.949,0.469)	0.508
	arm 3 vs. arm 1	0.11 (-0.392,0.611)	0.668	-0.023 (0.559,0.512)	0.932	0.315 (-0.605,1.234)	0.502	0.388 (-0.512,1.288)	0.398
	arm 4 vs. arm 1	0.338 (-0.076,0.753)	0.109	0.315 (-0.158,0.787)	0.192	0.72 (-0.115,1.555)	0.091	0.704 (-0.068,1.476)	0.074
	arm 5 vs. arm 4	0.188 (-0.274,0.651)	0.425	0.111 (-0.36,0.581)	0.645	-0.2 (-1.08,0.681)	0.657	-0.398 (1.193,0.397)	0.327
	arm 6 vs. arm 4	0.008 (-0.528,0.544)	0.977	-0.204 (0.763,0.355)	0.474	-0.635 (-1.52,0.251)	0.160	-0.98 (-1.914,-0.046)	**0.040**
5-to-8-year olds	arm 2 vs. arm 1	-0.066 (-0.547,0.414)	0.788	-0.192 (0.762,0.378)	0.509	-0.117 (0.749,0.516)	0.718	-0.576 (1.668,0.517)	0.302
	arm 3 vs. arm 1	0.245 (-0.344,0.834)	0.415	0.119 (-0.564,0.803)	0.732	0.698 (-0.033,1.428)	0.061	0.778 (-0.227,1.784)	0.129
	arm 4 vs. arm 1	0.016 (-0.532,0.563)	0.955	-0.09 (-0.644,0.465)	0.751	0.373 (-0.497,1.243)	0.400	0.187 (-0.651,1.025)	0.662
	arm 5 vs. arm 4	0.48 (-0.011,0.97)	**0.050**	0.422 (-0.1,0.944)	0.113	0.136 (-0.708,0.98)	0.752	0.128 (-0.72,0.975)	0.768
	arm 6 vs. arm 4	0.024 (-0.53,0.578)	0.932	0.021 (-0.643,0.686)	0.950	-0.143 (1.093,0.806)	0.767	-0.341 (1.229,0.547)	0.451

Significance testing to assess whether the prevalence of *S*. *haematobium* varied over time between study arms showed that gender (p<0.001) and age (p<0.001) had a significant effect on infection status with higher prevalence and proportion of those heavily infected seen in males and among the 9-to-12-year age group in Year 5 (Table 3 and S2 Table). Across study year, prevalence of infection significantly decreases over the five years (p<0.001) with no statistically significant effect across the study arms. For the SBT arms, Arm 4 (annual SBT) was used as the reference arm to assess the impact of treatment holidays on prevalence. There was no significant difference seen following holidays. The impact of four versus two treatments was also assessed with the GEE model, where four treatments over four years (for both CWT and SBT) had a significantly greater impact in reducing prevalence of infection (p<0.024) than two treatments over four years. There was no significant impact between the rounds of treatment and intensity of infection (S3 Table).

### Other age cross sections

In Year 1 and 5 of the study an additional 31,542 observations were recorded for first-year students (aged 5-to-8-years) and 50 adults (aged 20-to-55-years) (Table 4). At baseline, examination revealed that of the 7,456 first-year students, 62.8% were infected with *S*. *haematobium* and out of 4,253 adults, 44.5% were found to be infected. The proportion of heavily infected first-year students was higher compared to adults, with 19.3% and 7.1%, respectively. In Year 5 prevalence had fallen to 39.3% and 26.4% among first-year students and adults, respectively, and proportion of heavily infected had decreased to 12.8% and 5.6%, respectively.

**Table 4:** Summary of *S*. *haematobium* infection for years 1 and 5 by study arm (first-year students and adults).

Year	Arm	First year students (5-8-years)				Adults (20-55-years)			
		Number tested	Number infected (%)	Heavily infected (%)	Mean infection intensity	Number tested	Number infected (%)	Heavily infected (%)	Mean infection intensity
**1**	**1**	1,419	846 (59.6)	239 (16.8)	50.0	735	351 (47.8)	64 (8.7)	25.5
	**2**	1,148	751 (65.4)	239 (20.8)	66.1	615	328 (53.3)	60 (9.8)	33.5
	**3**	1,465	913 (62.3)	291 (19.9)	68.0	890	367 (41.2)	51 (5.1)	23.2
	**4**	1,296	765 (59.0)	189 (14.6)	42.0	652	210 (32.2)	33 (5.1)	16.6
	**5**	1,191	823 (69.1)	247 (20.7)	64.2	703	373 (53.1)	59 (8.4)	29.3
	**6**	937	586 (62.5)	234 (25)	59.3	658	265 (40.3)	33 (5.0)	15.2
	**Total**	7,456	4,684 (62.8)	1,439 (19.3)	58.1	4,253	1,894 (44.5)	300 (7.1)	23.9
**5**	**1**	2,251	808 (35.9)	262 (11.6)	46.6	1,151	247 (21.5)	49 (4.3)	20.2
	**2**	2,282	785 (34.4)	215 (9.4)	41.5	1,008	230 (22.8)	43 (4.3)	14.4
	**3**	2,069	893 (43.2)	337 (16.3)	84.8	1,181	287 (24.3)	64 (5.4)	27.8
	**4**	2,320	845 (36.4)	287 (12.4)	63.3	1,085	311 (28.7)	93 (8.6)	40.6
	**5**	2,205	1,085 (49.2)	369 (16.7)	77.5	1,084	357 (32.9)	71 (6.5)	34.1
	**6**	2,117	784 (37.0)	224 (10.6)	60.0	1,080	308 (28.5)	50 (4.6)	21.5
	**Total**	13,244	5,200 (39.3)	1,694 (12.8)	61.8	6,589	1,740 (26.4)	370 (5.6)	26.5
**Absolute difference in prevalence?**			-23.5				-18.1		
**Relative difference in prevalence?**			-37.4				-40.7		

Significance testing to assess whether the prevalence of *S*. *haematobium* differed between study arms across the first-year students and adults in Year 5 are shown in Table 3 and S4 Table. From the GEE prevalence models adjusted for age and sex, gender was a significant effect in first-students only (p<0.001) with males displaying a higher probability of being infected than females. Increasing age was also significantly associated with infection among first-year students, with the oldest respondents (8 years) being more likely to be infected than seven and six-year olds (p<0.001). There was no statistical association between age or sex with risk of infection in adults. For first-year students and adults, both the adjusted and unadjusted prevalence models show no significant difference in prevalence between the study arms at the end of the study.

**Fig 5:**
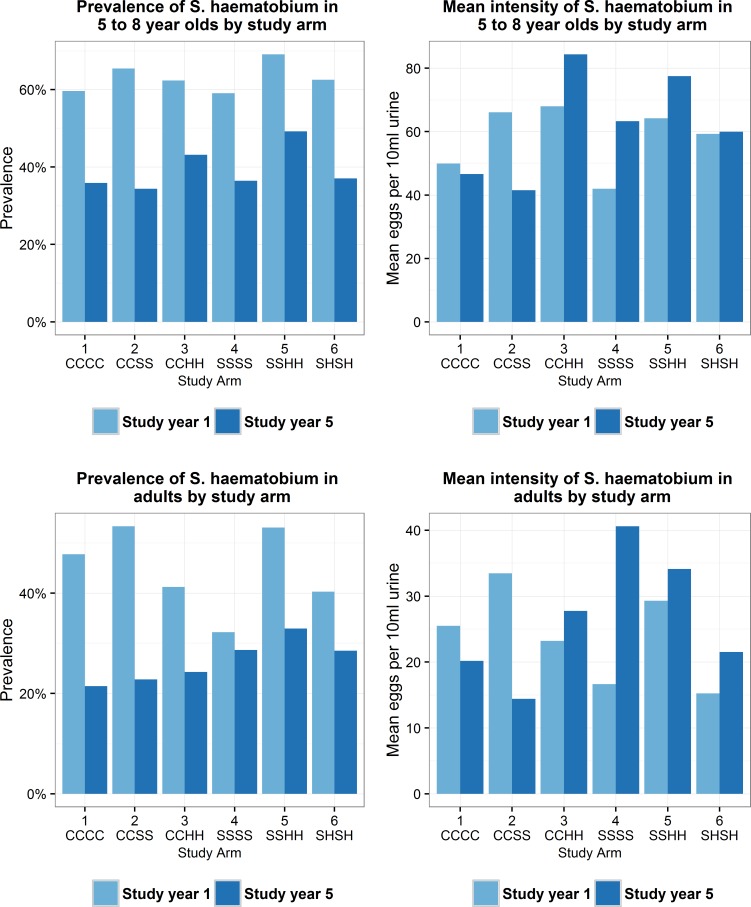
Prevalence of *S*. *haematobium* infection and mean intensity by study arm at baseline and Year 5 for first-year students (aged 5-8-years) and adult cross section. Each graph shows the change in prevalence of *S*. *haematobium* infection and mean intensity, at baseline and Year 5, separated by study arm. There was large reduction in prevalence in both first-year students and adults from baseline to Year 5 across all study arms. Mean intensity of infection decreased in first-year students and adults in arm 1 and 2 only. In the remaining study arms (3–6), mean intensity levels increase from baseline to Year 5 in both first-year students and adults. It appears therefore that although the number of individuals infected reduces over time, the burden of infection in infected individuals seem to be heavier. It is not clear whether individuals harbouring a high parasite burden in such highly endemic populations are being re-infected each year, are more likely to be non-compliant in terms of taking the treatment, or if the parasites have developed resistance to treatment.

Of particular interest is the statistically significant reduction in prevalence of infection for adults from Year 1 to 5 in the SBT arms (Fig 5). Adults did not receive treatment in these arms, however, the GEE prevalence model shows no significant difference in prevalence for adult’s focal comparison arms. This would suggest that SBT alone has an impact on disease transmission.

### Treatment coverage by strategy over time

Drug coverage was defined as the proportion of individuals who have ingested the PZQ [7]. The denominator is the estimated target population. The SBT coverage was determined by the percentage of school-aged children (5-12-years) that were treated, including those not enrolled in school. CWT coverage was determined by the total population treated using a denominator of the whole population based on the census carried out by the SCORE study in 2011. Coverage by study arm for each year is outlined in Table 5.

**Table 5:** Treatment numbers and percentage coverage by study arm for each year.

	SBT coverage			CWT coverage		
Study Arm	SAC treated	SAC total for treatment	% SAC treated	Total number treated	Total CWT for treatment	% total CWT treated
Year 1						
1 (cccc)	CWT given in Year 1 in Arms 1–3			20,284	70,495	28.8%
2 (ccss)				16,692	71,728	23.3%
3 (cshh)				19,687	50,559	38.9%
4 (ssss)	7,661	24,804	30.9%	8,139	SBT given in Year 1 in Arms 4–6	
5 (sshh)	7,039	24,022	29.3%	7,292		
6 (shsh)	6,421	24,297	26.4%	6,494		
Year 2*						
1 (cccc)	CWT given in Year 2 in Arms 1–2			31,505	70,495	44.7%
2 (ccss)				25,486	71,728	35.5%
3 (cshh)	7,998	13,609	58.8%	21,047	SBT given in Year 2 in Arms 3–5	
4 (ssss)	5,638	24,804	22.7%	6,478		
5 (sshh)	7,764	22,193	35.0%	8,186		
Year 3*						
1 (cccc)	CWT given in Year 3 in Arms 1			37,858	70,495	53.7%
2 (ccss)	7,239	21,518	33.6%	7,658	SBT given in Year 3 in Arms 2, 4 and 6	
4 (ssss)	543	966	56.2%	651		
6 (shsh)	6,617	24,297	27.2%	7,671		
Year 4*						
1 (cccc)	CWT given in Year 3 in Arms 1			91,530	70,495	129.8%
2 (ccss)	5,812	21,518	27.0%	5,977	SBT given in Year 3 in Arms 2 and 4	
4 (ssss)	5,648	24,804	22.8%	5,786		

*Arms with treatment holidays do not have treatment data in these years.

The nominal treatment coverage stipulated by the study protocol was coverage of more than 75% for both SBT and CWT [7], which did not occur in many communities due to suspected inaccuracies in denominator values. The results shown in Table 5 shows that SBT coverage remained consistently low over time across all arms. CWT coverage, however, did increase over time across all arms, with no significant difference seen between arms.

When coverage was put into the multivariate model looking at impact on changes of prevalence over time, there was no clear trend or significant relationship with impact on infection.

### Financial costs of different treatment strategies

Delivery costs (defined as the cost per person treated minus the drug costs) was $0.31 for SBT and $0.36 for CWT. The relative costs by each activity and input are summarised in Table 6. Our cost-analysis does not consider unpaid days of labour for individuals such as teachers, or other costs related to the SCORE field work. When the financial costs were broken down by activity, there was substantial cost saving for SBT for supervision and monitoring as this was carried out by the SCORE field teams during the prevalence survey. For CWT on the other hand, additional costs were incurred as supervisors were sent back to the villages post-survey to supervise and collect treatment registers.

**Table 6:** Financial costs of treatment strategy.

**Cost by Activity**	**CWT**	**SBT**
Training	$236.79	$133.23
Advocacy	$290.00	$290.00
Supervision/Monitoring	$102.31	$3.60
Drug Distribution & Coverage	$691.19	$302.30
**TOTAL**	**$1,320.30**	**$729.13**
**Cost by Input**	**CWT**	**SBT**
Per diem Ξ	$818.00	$365.00
Vehicle Maintenance	$268.00	$268.00
Fuel	$132.47	$57.75
Materials*	$101.83	$38.38
**TOTAL**	**$1,320.30**	**$729.13**
**Total cost per person treated**	**CWT**	**SBT**
Total Cost	$1,320.30	$729.13
Cost/Village	$264.06	$159.23
Average number treated in Year 4	3661	874
**Cost/Person treated**	$0.36	$0.31

Ξ Per diems include national (coordinators and technical staff), district and regional health and educational staff, and community level staff including community drug distributors, teachers, community health workers etc.

*Materials include Phone cards, information/education/communication posters, treatment registers, dose poles etc.

According to input costs, there was a greater cost in per diems due to additional costs of community drug distributors salary and incentives (where one distributor was recruited for every 500 people in the village), whereas teachers were not paid extra in the schools but given incentives (a t-shirt) only. Fuel costs were also higher for CWT since the teams needed to re-visit the communities a second time to collect any left-over drugs and the treatment registers. In SBT the treatment was done on the same day as the prevalence survey and the registers taken. The additional material costs for CWT were incurred due to additional treatment registers printed for larger numbers being treated.

## Discussion

In Cabo Delgado, Mozambique, *S*. *haematobium* infection was found prevalent throughout all 149 schools in the study, where many schools had 90% baseline prevalence (Fig 2). The primary outcome of the study was to examine the change in infection among 9-to-12-year olds between specified study arms. Our findings showed a significant decrease in *S*. *haematobium* infection in this age group from a mean prevalence of 66.4% in Year 1 (of which 22.5% were heavily infected) to 42.5% in Year 5 (when 13.4% were heavily infected). When comparing the impact of treatment strategy each year, the only significant response to study arm was seen between annual SBT (arm 4) and two years of SBT followed by two consecutive treatment holidays (arm 5), which resulted in a higher final prevalence than when there were no treatment holidays. There were no significant differences in Year 5 prevalence for the other treatment comparisons. The general uptrend in prevalence and intensity of *S*. *haematobium* infection shown after two subsequent holiday years, compared with one-year post-treatment, demonstrates rebound of *S*. *haematobium* if PC is interrupted. These findings of significant risk for re-emergence of infection if treatment is suspended supports previous schistosome infection modelling [30, 31].

Other studies have shown that the highest prevalence and intensities of infection occurred in males and among the 9-to-12-year age group, with infection decreasing in adulthood [32–36]. Despite a higher starting prevalence in adolescent males, there was no statistically significant difference in response to treatment by gender or age across the study arms. There is a need to explore further whether boys in these regions are more susceptible to subtle morbidity and impaired growth, mental or physical performance than girls.

The limitations of the current diagnostic methods for urogenital schistosomiasis have been demonstrated elsewhere, where the current ‘gold standard’ of urine filtration has been shown to be insensitive, especially in areas with low endemicity, and egg secretion varies from day-to-day [37, 38]. However, in the context of Cabo Delgado where the prevalence and intensity of infection even in Year 5 remained high, it is likely that infections were not underestimated. Furthermore, aim of this study is to look at relative reduction in infection over time and given that microscopy was done consistently over the years, the trend in change is believed to be valid.

As shown in the SatScan spatial scan, there is a decrease in the number of high prevalence spatial clusters from two western clusters at baseline to a single and less scattered central cluster in Year 5. A possible explanation for this is that as the prevalence of infection decreases over time, heterogeneities in the force of transmission by geography or social group become more visible. Such geographical clustering has also been seen in other SCORE projects in Kenya [39]. Further research is therefore necessary to understand how transmission dynamics change by space and social group as prevalence reduces, particularly among more resilient places/groups, and therefore how to adapt interventions accordingly.

There is a growing body of evidence demonstrating the burden of infection and morbidity in adults, as well as their potential role in sustaining transmission [14, 30, 31, 40–42]. Indeed, the results here show that in arms 3–6 mean intensity levels increase from baseline to Year 5 in adults. It appears that although the number of individuals infected reduces over time, the burden of infection in those infected seem to be heavier. It is not clear whether individuals harbouring a high parasite burden in such highly endemic populations are being re-infected each year, are non-compliant in taking the treatment, or if the parasites have developed resistance to treatment. This implies a greater need to include adults in schistosomiasis PC treatment programmes in some endemic settings. The SCORE study is therefore crucial as it investigates the impact of CWT in reaching more adults. The findings from Mozambique, however, have demonstrated a significant reduction in prevalence of schistosomiasis in adults even in the SBT arms, implying transmission in the community has been decreased, even where only school children have been treated. These results support previous findings that demontrates the potential benefit of switching to community-wide mass treatment is highly variable in different contexts [43, 44]. The delivery cost for SBT and CWT in this SCORE study is summarised in the Table 6 where $0.31 was calculated for SBT and $0.36 for CWT per person treated. Although this difference is small on an individual basis, in 2016 the Mozambique Ministry of Health treated around 8.5 million people and therefore these results therefore have significant logistical and cost-saving implications for a national control programme as they challenge the justification for CWT. Furthermore, a crude analysis of the relative cost per person treated by percentage reduction in infection for each study arm implies that even though two treatments over five years reduces prevalence less than four treatments, two treatments has a greater percentage reduction by relative cost.

Village-level data, both in communities and at the school, were collected about water sources, sanitation, and hygiene (WaSH) issues through key-informant interviews. The baseline results from the WaSH survey have been published elsewhere [21]. In brief, among our study population, use of open surface water was widely reported for washing and bathing, and although pit latrines were reported to exist in most communities, open urination was widely practised. Furthermore, nearly all the communities in this study rely on subsistence farming whereby a large proportion of villagers spend months at a time sleeping in tented accommodation whilst working in the ‘machambas’ (fields), with no access to sanitation. Despite, such reported high-risk behaviour when the WaSH data was incorporated into the multivariate model, there were no significant findings associated with change in prevalence and intensity of infection across the study arms.

The most plausible explanation for why more intense treatment approaches did not result in a better impact on infection reduction was most likely due to poor treatment coverage. The nominal treatment coverage stipulated by the protocol was more than 75% for both SBT and CWT, which did not occur in many communities. A qualitative Knowledge Attitudes and Practices (KAP) survey was carried out in Year 3 to understand the challenges to achieving sufficient coverage, this showed contextual issues extraneous to the study that included extremely low school attendance (around 30% in majority of communities), high proportion of individuals (including school-aged children) working in fields or mines during the day, acute flooding which proved challenging to the CWT, severe political unrest and subsequent mistrust in any health-related programmes following cholera outbreaks, and poor study sensitisation in some areas. Although several days were spent by the survey team in each community to mobilise inhabitants, many communities were sparsely populated for several weeks whilst people were living in their fields or away in the mines for extended periods of time, which also highlighted the issue of unreliable denominator data. Nevertheless, there was an increase in CWT coverage over time that was due raised awareness of the programme and less trepidation in taking the treatment as the programme progressed, distribution of t-shirts among key community individuals in Year 3 and in Year 4 food was provided free-of-charge with treatment to help increase treatment coverage. Other studies have shown that provision of a pre-treatment snack can result in reduced side-effects as well as decreased prevalence of schistosomiasis [45]. These strategies proved effective with regards to community treatment coverage, but less impact on school treatment coverage. Such challenges in attaining good coverage is a reality for national control programmes, particularly in the light of poor school attendance [46]. There it is even harder to achieve than in a study setting such as ours, where there are generally more time and resources allocated to sensitising and motivating communities to participate.

### Conclusions

The results obtained show that Cabo Delgado province in Northern Mozambique is highly endemic for urinary schistosomiasis. Although PC was successful in reducing the burden of active infection in all study arms, a more intense treatment approach, such as annual CWT, did not have as much impact as expected. This may be due to extremely high starting prevalence and intensity in the study area, with frequent exposure to reinfection [47]. It also may be related to poor treatment coverage and the challenge of performing a long-term study in a resource-poor setting, which may not be valid in other locations. These findings highlighted the need to complement mass drug administration with extensive public health education and behavioural modifications to reduce the risk of reinfection, and to consider the use of vector control through mollusciciding in areas where compliance to treatment proves to be challenging [38, 48]. There is a need to explore further those individuals harbouring a high parasite burden in such highly endemic populations to understand whether they are being re-infected each year, are more likely to be non-compliant in terms of taking the treatment, or if the parasites might have developed resistance to treatment. The results of this study have also highlighted the challenge of achieving high treatment coverage especially with such highly migrant communities, specifically acquiring accurate population figures, and the importance of independent coverage surveys that do not rely on denominator values to accurately evaluate the impact of a treatment programme. Furthermore, more frequent treatment had a greater impact on prevalence of infection when arms were grouped by number of treatments, however, the costs efficiency was greater in arms only receiving two treatments. Finally, the significant reduction in prevalence of schistosomiasis among adults even in the SBT Arms implying transmission in the community has been decreased, has significant logistical and cost-saving implications for a national control programme as they challenge the justification for CWT.

## Supporting information

S1 Fig Spatial clusters of high prevalence villages calculated in SatScan. Spatial clusters of high prevalence villages calculated in SatScan show a decrease in the number of disease clusters from baseline (Year 1) to Year 5, and the spatial extent of disease became less scattered over time. Each point represents an individual village whereby all communities in red have an overall prevalence of infection that is greater than the overall mean infection prevalence across all villages at that time point.
(TIFF)

S2 Fig Prevalence of *S*. *haematobium* by individual villages over time by study arm. Prevalence of *S*. *haematobium* by individual villages, shown by different coloured points, over time by study arm. The black line is the mean village prevalence, by study arm, at each year. In treatment holidays shown in Arms 3, 5 and 6, there was no data collected and therefore no data points for this year.
(TIFF)

S1 Table Summary of *S*. *haematobium* infection rates for each study year and study arm (9-to-12-year-old cross section only). Village-level *S*. *haematobium* prevalence, prevalence of heavy infection, and mean intensity for each study year from baseline to Year 5, by study arm among 9-12-year old cross section only.
(TIFF)

S2 Table GEE prevalence- and intensity-adjusted parameter estimates (9-12-year olds only). (TIFF)

S3 Table GEE logistic model to assess differences in prevalence and intensity of *S*. *haematobium* infection between four versus two rounds of treatment in 9-12-year olds. (TIFF)

S4 Table GEE logistic model to assess differences in prevalence and intensity of *S*. *haematobium* infection in first-year students only. (TIFF)
