# Supplementary figures and images for: Assessing the benefits of five years of different approaches to treatment of urogenital schistosomiasis: A SCORE project in Northern Mozambique
Source: PLoS Negl Trop Dis. 2017 Dec 8;11(12):e0006061. doi: 10.1371/journal.pntd.0006061 (PMC5745126; doi:10.1371/journal.pntd.0006061)

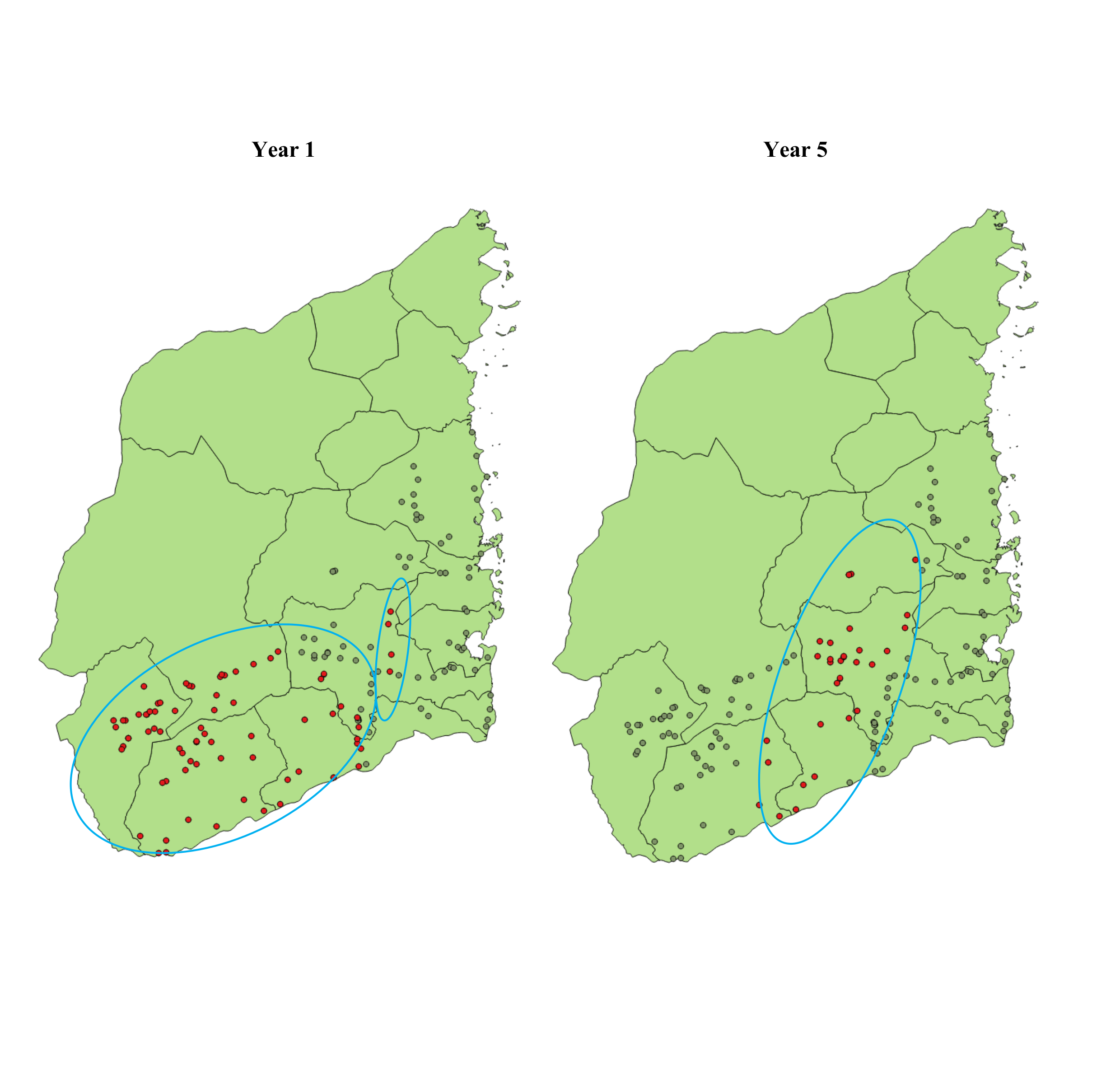

Supplement: S1 Fig — Spatial clusters of high prevalence villages calculated in SatScan show a decrease in the number of disease clusters from baseline (Year 1) to Year 5, and the spatial extent of disease became less scattered over time. Each point represents an individual village whereby all communities in red have an overall prevalence of infection that is greater than the overall mean infection prevalence across all villages at that time point. (TIFF) [file pntd.0006061.s001.tiff]

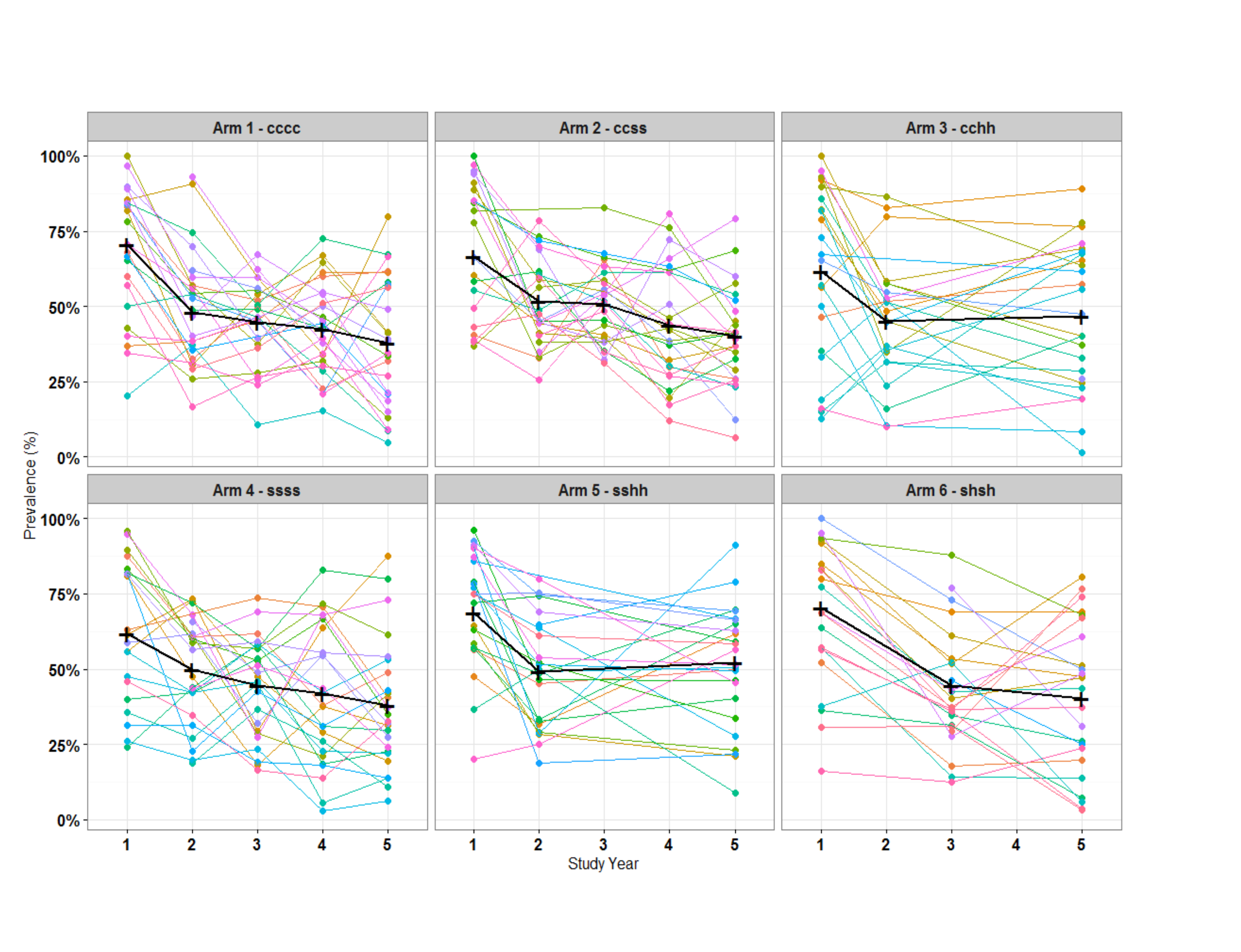

Supplement: S2 Fig — Prevalence of S. haematobium by individual villages, shown by different coloured points, over time by study arm. The black line is the mean village prevalence, by study arm, at each year. In treatment holidays shown in Arms 3, 5 and 6, there was no data collected and therefore no data points for this year. (TIFF) [file pntd.0006061.s002.tiff]

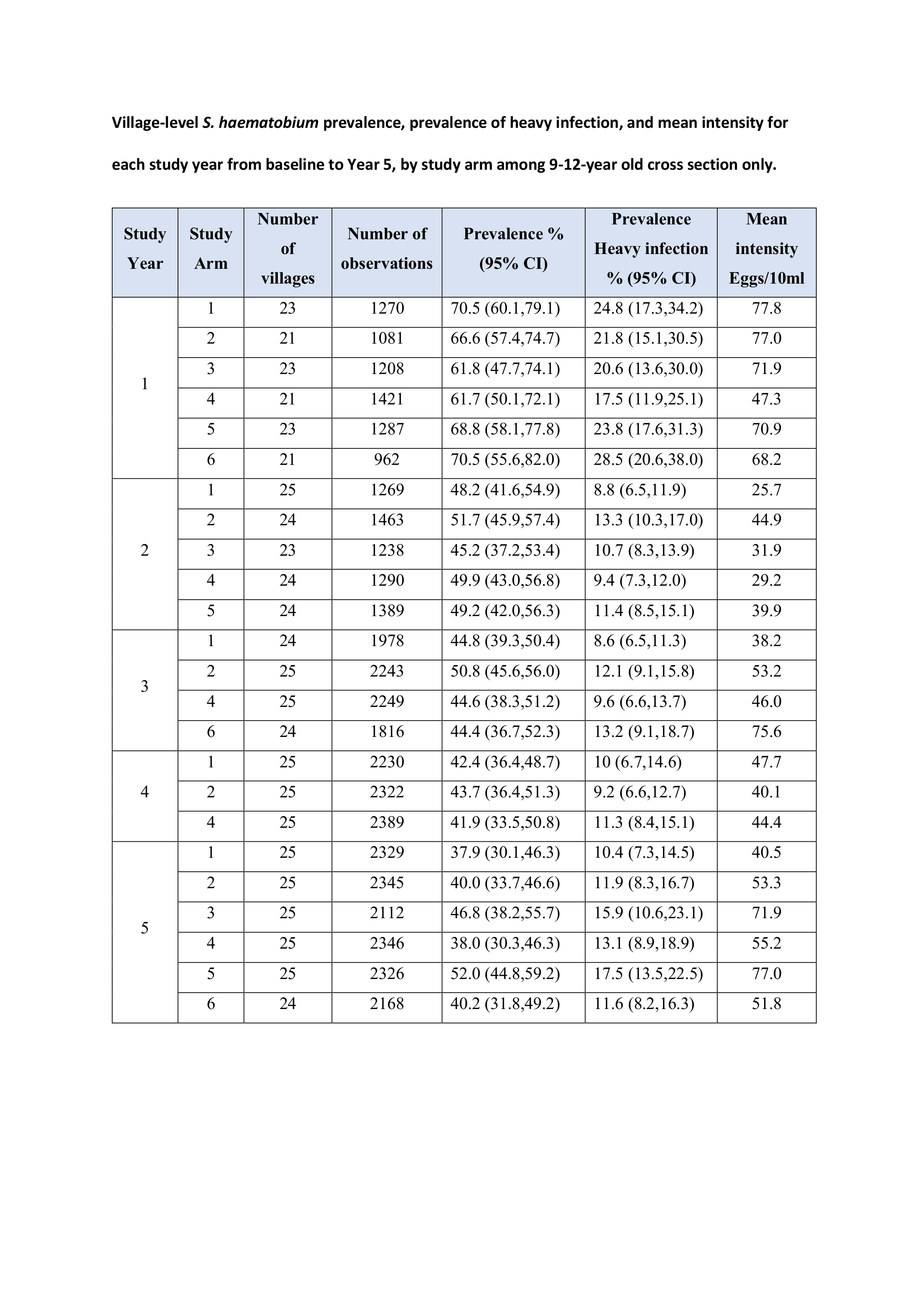

Supplement: S1 Table — Village-level S. haematobium prevalence, prevalence of heavy infection, and mean intensity for each study year from baseline to Year 5, by study arm among 9-12-year old cross section only. (TIFF) [file pntd.0006061.s003.tiff]

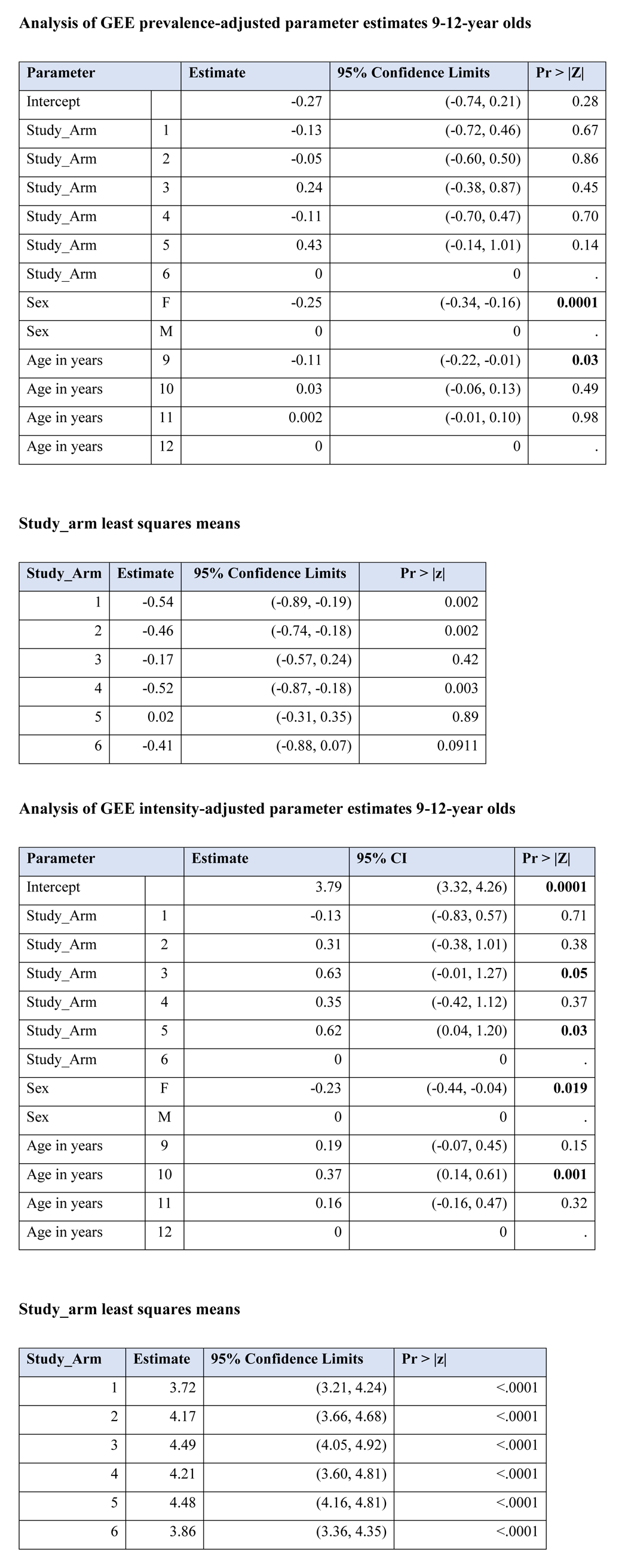

Supplement: S2 Table — (TIFF) [file pntd.0006061.s004.tiff]

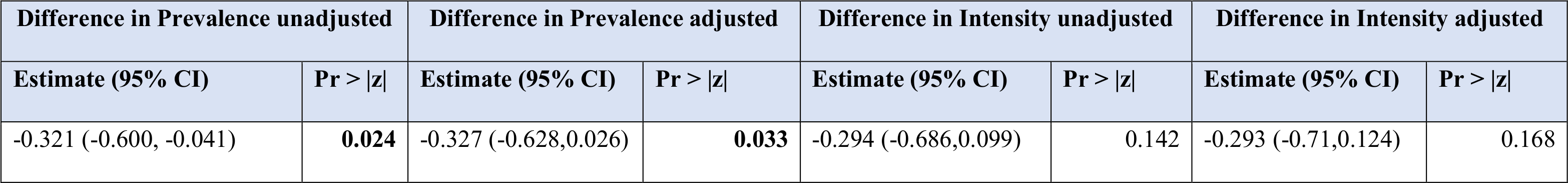

Supplement: S3 Table — (TIFF) [file pntd.0006061.s005.tiff]

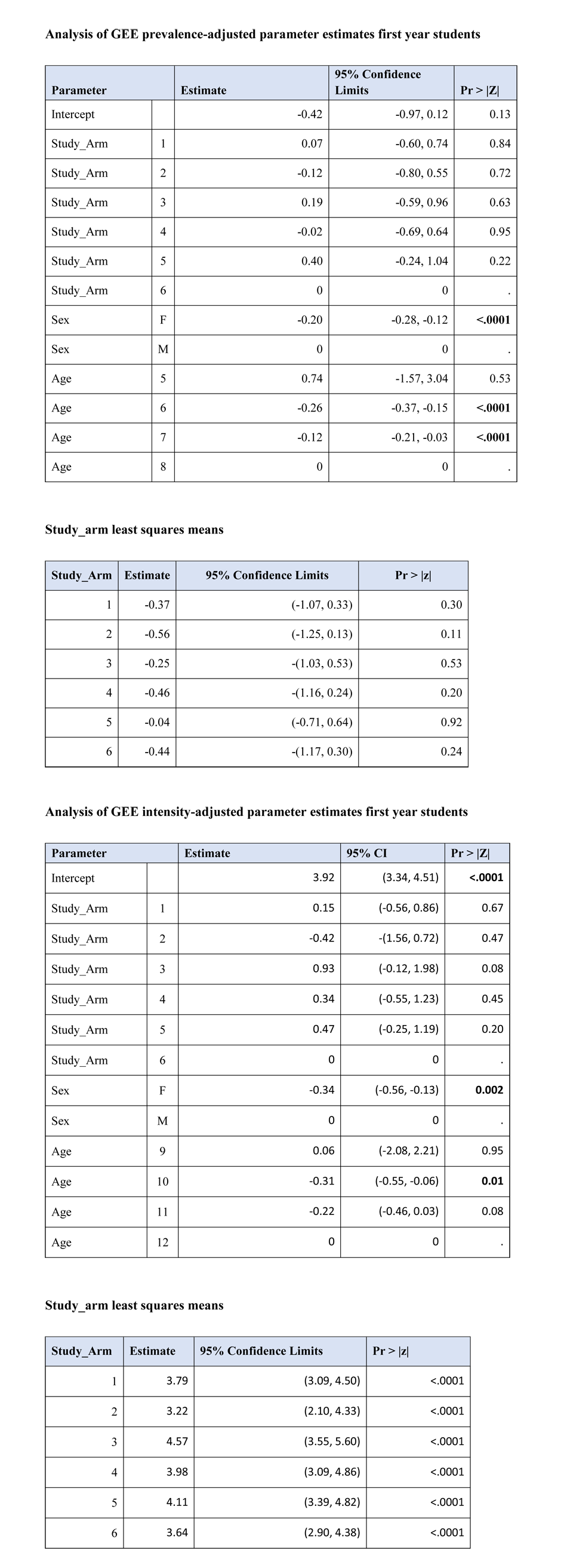

Supplement: S4 Table — (TIFF) [file pntd.0006061.s006.tiff]
